# Better treatment option in chronic superficial femoral artery occlusive disease: comparison of methods (meta-analysis)

**DOI:** 10.15171/jcvtr.2019.37

**Published:** 2019-08-28

**Authors:** Vladimir Starodubtsev, Andrey Karpenko, Evgeniy Lenko, Pavel Ignatenko

**Affiliations:** Siberian Federal Biomedical Research Center, Ministry for Public Health Care of Russian Federation, Novosibirsk, Russian Federation

**Keywords:** Excisional Atherectomy, Endovascular Procedure, Superficial Femoral Artery, Remote Endarterectomy, Chronic Occlusive Disease

## Abstract

***Introduction:*** The objective is to evaluate the frequency of primary obstruction events (PrO) during one-year follow-up after performing excisional atherectomy with the SilverHawk/TurboHawk atherectomy device (S/TH) or remote superficial femoral artery endarterectomy (RSFAE) in patients with the chronic superficial femoral artery occlusive disease (СSFAOD).

***Methods:*** We included all randomized clinical trials (RCTs) and not-RCTs concerning the treatment of patients with СSFAOD after S/TH and RSFAE without duration.

***Results: *** Twenty-nine items (1990-2017) were discovered; 27 articles on the levels of evidence were included in qualitative synthesis; 9 studies (meta-analysis) were included in quantitative synthesis. The results of 2762 patients’ treatment were summed up in our analysis (1422 patients S/TH; 1340 patients RSFAE). All included reports were at low risk of bias. According to the criterion "frequency of PrO" during one-year follow-up, the pooled Hazard Ratios indicate significant favours of S/TH if compared it with RSFAE (HR= 0.66 (0.57 to 0.76, P < 0.00001), I2 = 9%).

***Conclusion: *** Our study showed that S/TH with the SpiderFX device (distal embolic protection) are safe and effective treatment option for short lesion (<15 cm) in patients with СSFAOD. The usage of S/TH methods significantly reduced number of PrO if compared it with RSFAE. In long-segment lesion (>15 cm) in patients with СSFAOD, RSFAE may be considered better than an endovascular procedure. But still it is necessary to conduct well-planned randomized studies to determine effectiveness and safety of the compared methods (S/TH and RSFAE) in patients with long-segment lesion (>15 cm).

## Introduction

Excisional atherectomy with the SilverHawk/TurboHawk^TM^ atherectomy device is one of the first atherectomy methods of peripheral arteries disease treatment. Directional atherectomy (TurboHawk and SilverHawk, Medtronic/Covidien/ev3) is advised to treat lesions in the infrainguinal superficial femoral (SFA), popliteal artery (PA) and below-the-knee vessels.^1^ The device fulfills two functions due to its elements: a rotating blade removes plaque while a nose cone gathers it away. TurboHawk^®^ Peripheral Plaque Excision System (TurboHawk Catheter and ev3 Cutter Driver, Plymouth, MN, USA) design focuses on the treatment of primary and restenotic atherosclerotic lesions, both calcified and noncalcified, placed in native peripheral arteries. The TurboHawk^TM^ Plaque Excision System is Covidien’s most advanced directional atherectomy platform to safely treat peripheral arterial disease above and below the knee. When the TurboHawk catheter is used in the case of hard, complex calcified lesions, it should be accompanied by the SpiderFX™ Embolic Protection Device to eliminate the distal embolization risk.^2^

The objective of this review is to assess the frequency of primary obstruction events (PrO) during one-year follow-up after performing excisional atherectomy with the SilverHawk/TurboHawk atherectomy device (Group S/TH) or remote superficial femoral artery endarterectomy (Group RSFAE) in patients with the chronic superficial femoral artery occlusive disease (СSFAOD).

*Hypothesis:* S/TH is an effective method of treatment of patients with СSFAOD (compare with RSFAE).

## Materials and Methods

### Criteria for considering studies for this review

We used the derivate of PICO-format for best formatting clinical questions in the used search strategy to identify relevant articles.

### Types of participants

Adults (older than 18 years), with *СSFAOD* were observed in our research.

### Types of interventions

Excisional atherectomy with the SilverHawk/TurboHawk^TM^ atherectomy device (endovascular procedure with SilverHawk^TM^/TurboHawk^TM^, Group S/TH) and remote superficial femoral artery endarterectomy (Group RSFAE, Controls) were evaluated.

### Primary outcomes

Primary obstruction events (restenosis, occlusion) during one-year follow-up

#### Definitions

Target lesion (or limb) revascularization (TLR) is defined as a re-intervention performed for ≥50% diameter stenosis (confirmed by angiography) within ±5 mm proximal and/or distal to the area of previously treated luminal narrowing following documentation of recurrent clinical symptoms or objective measures of restenosis.^3^

### Types of studies

**Figure 1:**
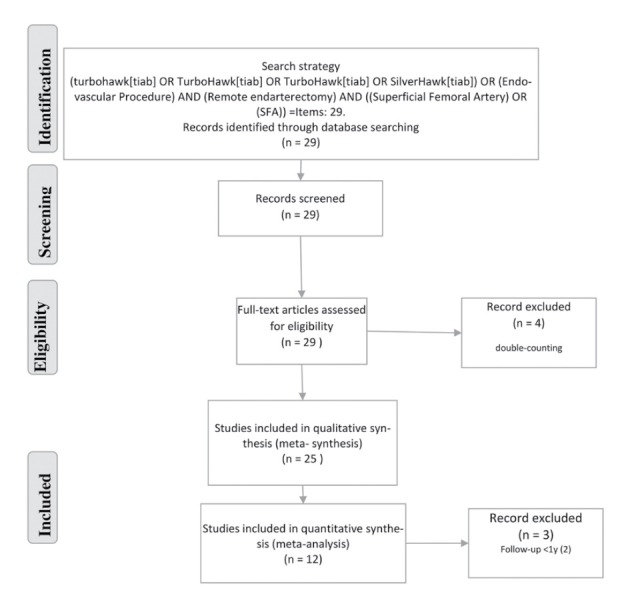
Flow-chart of processes of systematic search and selection of studies for synthesis.

We included all studies, reporting about *PrO* in patients with *СSFAOD* after *S/TH* and *RSFAE*, regardless of their design. For review we retrieved articles with following report characteristics: English- and non-English language (Figure 1). ClinicalTrials.gov was a source to assure the identification of applicable ongoing researches.

### Assessment of quality included studies

Each study, included in this meta-analysis, was evaluated by authors independently, the level of evidence for each study was heuristically rated with the Oxford Centre for Evidence-Based Medicine 2011 levels of evidence on a scale of 1 to 5.^4^ Each regarded study was assorted according to this schema.^5^

### Assessment of risk of bias (RoB) in included studies

The chosen items were assessed by the authors independently using the RoB assessment tool. In included studies for assessment of RoB, we applied RoBANS, risk of the bias assessment tool.^6^

### Measures of treatment effect

We expressed dichotomous outcome results, if the “momentary risk” (“hazard”) of PrO for a certain time interval was possible to evaluate and/or if the article contained “time-to-event” data, we expected hazard ratio (HR) with 95% CI.

### Assessment of heterogeneity

We applied the I^2^ statistic using Pearson’s chi-squared test to check out heterogeneity (alpha of 0.05 for statistical significance). We regarded I^2^ values of 25%, 50%, and 75% to correspond to low, medium, and high levels of heterogeneity.

### Data synthesis

All assessments were carried out with Review Manager 5.3.5 (RevMan 5.3.5, Nordic Cochrane Center, Copenhagen, Denmark). We used the Generic Inverse Variance method (GIVM) to summate the calculated HR.

## Results

By searching through databases (1990-2017) mentioned above, 29 items of interest were discovered but only 27 were included in qualitative synthesis. The quality of the studies included was reviewed with the help of the Oxford Centre for Evidence-Based Medicine 2011 Levels of Evidence and, moreover, each study was evaluated concerning their findings (the number of included articles on the “Levels of Evidence Oxford 2011”, LoE: 1/2/3/4/5 =4/2/10/5/6, respectively). In quantitative synthesis 9 studies (meta-analysis) were included (LoE: 1/2/3/4/5 = 0/2/3/1/0, respectively). The results of 2762 patients’ treatment were summed up in our analysis (Group S/TH (1422 patients); Group RSFAE (1340 patients).

In the meta-synthesis and meta-analysis we included the studies containing the best evidence and the data (Table S1, Table S3, see online Supplementary file 1).

The main conclusions based on the best evidence from studies were included in the Meta-synthesis and Meta-analysis (Tables S2 and S4, respectively; online Supplementary file 1 ).

### Level of Evidence 1

It has been proved that an Endovascular-First Approach is the first choice option in the majority of stenoses or occlusion lesions in patients with СSFAOD. After successful endovascular interventions all patients should be treated with single antiplatelet therapy. RSFAE is an effective minimally invasive treatment but, in the case of restenosis (>50%), an operator is to perform percutaneous transluminal angioplasty (PTA) to reinforce long-term patency.^7-10^

### Level of Evidence 2

After RSFAE primary, assisted primary, and secondary patencies are evidently higher than after an endovascular treatment.^11^ If the saphenous vein is not usable, RSFAE should be regarded as a better choice, because it is mini-invasive and prosthetic graft material cannot be necessarily used.^12^

### Level of Evidence 3

Minimally invasive interventions and their broad usage let scientists and practical surgeons look at RSFAE from a different angle. RSFAE is accepted as a primary option for the treatment of long-segment (>15 cm) SFA occlusive disease, while directional atherectomy (DA) is regarded as an attractive treatment option, improving luminal diameters without stents. To treat moderate and severely calcified lesions in SFA and/or PA it is quite safe and effective to apply S/TH when a distal embolic protection device is used.^13-20^

### Level of Evidence 4

Werner-Gibbings et al and Rosenthal et al present that the treatment of infrainguinal arterial steno-occlusive disease is implemented by DA as it is an effective endovascular option for short segment occlusive lesions of the femoral artery, especially accompanied by intraoperative ultra-sonography (USG).^21^

### Level of Evidence 5

In the case of short SFA lesions, percutaneous transluminal angioplasty (PTA) is the first option to select, but longer lesions bias patency rates lower. The combination of PTA and nitinol stent placement improves patency rates significantly. RSFAE with the stent being a hybrid technique is performed to remove a plaque from SFA with adjunctive stenting of the distal SFA.^1,22^

### Clinical bottom line

Endovascular-first approach with nitinol stent placement is recommended in the majority cases in the treatment patients with СSFAOD. All patients are recommended to receive a single antiplatelet therapy. S/TH methods with distal embolic protection are considered safe and effective in the endovascular treatment of moderate and severely calcified lesions in SFA. Besides S/TH are an effective treatment option for short lesions (<15 cm) in patients with СSFAOD. In cases of a long lesion (>15 cm), RSFAE is regarded as a more preferable procedure than endovascular interventions. In the case of restenosis (>50%) after RSFAE, the improvement of long-term patency is achieved by PTA. If the saphenous vein is not applicable, RSFAE might be regarded as an adequate treatment option.

### Meta-analysis

**Figure 2:**
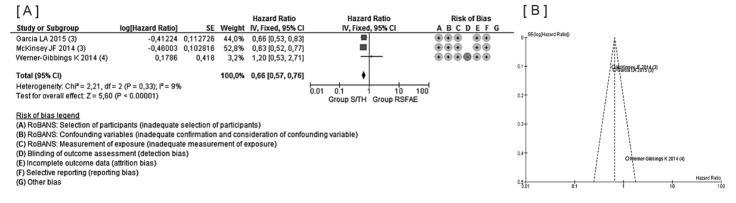
**(A)** Forest plot of comparison: “Primary obstruction events during one-year follow-up” (Group S/TH vs Group RSFAE). **(B)** Funnel plot.

Meta-analysis was performed by comparing two groups: Group S/TH and Group RSFAE. Group S/TH consisted of 1422 patients whose data were taken from 3 non-randomized studies. In Group RSFAE 1340 patients were included; the treatment information was taken out of 2 randomized trials and 4 non-randomized studies. Summing up, 9 studies were included (2762 patients) in quantitative synthesis and our indirect meta-analysis showed a significant reduction of *PrO* in Group S/TH during a one-year follow-up: HR 0.66 (0.57 to 0.76, *P* < 0.00001), I^2^ = 9%. No evidence of publication bias was provided by funnel plot (Figure 2).

### Summary (the main result)

*Clinical question*: does *S/TH* reduce *PrO* in patients with *СSFAOD*?

*Evidence-based answer*: Yes, it does, because patients from Group S/TH suffered fewer cases of PrO than patients of Group RSFAE (9 studies, 2 high quality & 7 lower, heterogeneous, inconsistent (I^2^=9%), HR 0.66 (0.57 to 0.76, *P* < 0.00001) 792 out of 2762 people experiencing PrO). The results of our meta-analysis support points of view expressed in some single researches,^23,24^ but it depends on all studies being found and there is an intrinsic bias towards “significant” findings,^25^ so to achieve more reliable and indisputable conclusion the performance of a well-planned randomized controlled blind trial is necessary.

*Risk of bias for reported outcomes*: 9 reports were evaluated for risk of bias for outcomes. All of them, 9 (100%), were considered to be at low risk of bias (for confounding factors).

## Discussion

PTA is the first choice in short SFA lesions, but patency rates decrease with longer lesions. However, when PTA is combined with nitinol stent placement, patency rates are significantly improved. Autologous venous conduit is still considered the “gold standard” for treatment of long occlusive SFA lesions. RSFAE with the stent is a hybrid surgical and endovascular technique that is useful for debulking plaques from SFA with adjunctive stenting of the distal SFA. There are multiple endovascular options to achieve percutaneous revascularization in patients with СSFAOD. The essential problem associated with these techniques is the predictable compromise of the initial result by neointimal hyperplasia. An alternative to forceful displacement techniques is the use of DA with the addition of low-pressure angioplasty or stent deployment as needed. Currently, DA is performed using the SilverHawk Plaque Excision System (FoxHollow, Red-wood City, CA).^26-28^

Nowadays it is known, in patients with СSFAOD, RSFAE did not reduce the expected HR of PrO, compared with S/TH. Conversely, S/TH was not associated with a significantly higher HR of PrO. Endovascular treatment with nitinol stent placement is recommended in the majority cases of steno-occlusive lesions in patients with СSFAOD; then all patients should receive at least single antiplatelet therapy. S/TH methods with the SpiderFX device (distal embolic protection) are safe and effective in the endovascular treatment of moderate to severely calcified lesions in the SFA. S/TH methods are an effective treatment option for short lesion (<15 cm) in patients with СSFAOD. In cases of long lesion (>15 cm) in patients with СSFAOD, RSFAE should be considered better than ENDO. PTA must be performed for improving long-term patency in cases of restenosis (>50%) after RSFAE. If the saphenous vein is not applicable, RSFAE should be performed as it is less invasive and prosthetic graft material can be avoided.^10,12^

But data of S/TH’ impact on PrO, and other adverse effects were limited to single-arm studies, it is necessary to compare S/TH and RSFAE for preventing PrO in patients with СSFAOD. Evidence about the benefits of S/TH procedure remains inconclusive: clinical outcomes, such as PrO, were not investigated in any included RCTs and are therefore still poorly applicable in clinical practice. Although the study of heterogeneity was not feasible due to the lack of studies included in each analysis, we can assume that the differences between individual studies design can represent one of the main reasons for this phenomenon. Procedural methods were also heterogeneous among the studies. The latest data show maximum treatment efficiency after RSFAE, but the lack of standardized methods in RSFAE for improving PrO may interfere with the reliability of comparisons between studies. Therefore, the “other bias”, the technical bias should be considered in future trials as a possible reason for the lack of response in many patients, and reliable markers to confirm the successful supports of S/TH.

As a result of meta-analysis, we gained new knowledge: the cumulative evidence is now conclusive that (1) the addition of excisional atherectomy with the SilverHawk/TurboHawk^TM^ atherectomy device (endovascular procedure with SilverHawk^TM^/TurboHawk^TM^) instead of RSFAE significantly reduces the PrO during a one-year follow-up among patients with СSFAOD. (2) It is necessary to conduct well-planned randomized studies with sufficient statistical power to determine the usefulness of the compared methods for improving PrO during a one-year follow-up in patients with СSFAOD.

Points of the strength of this review are represented by a systematic search of electronic databases, and data extraction, analysis quality, and RoB assessment completed independently by authors, according to current methodological standards.

All non-randomised studies included in quantitative synthesis (100%) were low level RoB because of blinding and others biases. The main limitation is represented by the data obtainable from the included studies. Studies were mainly focused on small populations and short treatment periods.

Recent studies^23,24^ suggest that S/TH is protective of PrO in patients with СSFAOD, but it depends on all studies being found and there is an intrinsic bias towards “significant” findings.^25^ The authors confirmed the current lack of evidence supporting the widespread use of this procedure in clinical practice, advocating for future clinical trials with a longer observation time.

## Conclusion

Our study showed that the S/TH with the SpiderFX device (distal embolic protection) are safe and effective treatment option for short lesion (<15 cm) in patients with СSFAOD. The usage of S/TH methods significantly reduced number of PrO if compared it with RSFAE.

In long-segment lesion (>15 cm) in patients with СSFAOD, RSFAE may be considered better than an endovascular procedure. But still it is necessary to conduct well-planned randomized studies to determine effectiveness and safety of the compared methods (S/TH and RSFAE) in patients with long-segment lesion (>15 cm).

## Competing interests

None declared.

## Ethical approval

The study was approved by the Local Ethics Committee of Siberian Federal Biomedical Research Center.

## Funding

This research received no specific grant from any funding agency in the public, commercial, or not-for-profit sectors.

## Supplementary Materials


Supplementary file 1 contains Tables S1-S4.
