# Supplementary material for: Better treatment option in chronic superficial femoral artery occlusive disease: comparison of methods (meta-analysis)
Source: J Cardiovasc Thorac Res. 2019 Aug 28;11(3):224–9. doi: 10.15171/jcvtr.2019.37 (PMC6759610; doi:10.15171/jcvtr.2019.37)
Supplement: Supplementary file 1 — contains Tables S1-S4. [file jcvtr-11-224-s001.pdf]

Table S1. The results of the data extraction from studies included in the qualitative –synthesis (total **27 studies**; Level of Evidence, Oxford 2011 (LoE: 1/2/3/4/5), = **4/2/10/5/6**, respectively)

First author, date, country of research, study type (and LoE)	Patient group	The outcomes investigated	Key results	PMID:
Akkus NI 2014; USA; (5)	Atherectomy is a procedure which is performed to remove atherosclerotic plaque from diseased arteries. Atherosclerotic plaques are localized in either coronary or peripheral arterial vasculature and may have different characteristics depending on the texture of the plaque.	Atherectomy has been used effectively in treatment of both coronary and peripheral arterial disease.	Atherectomy devices are designed differently to either cut, shave, sand, or vaporize these plaques and have different indications. In this article, current atherectomy devices are reviewed.	25565904
Park YS 2017 Seoul, Korea retrospectively (4)	Patients who underwent DA from January to December 2014 were reviewed retrospectively. 20 lesions from 14 patients with femoral artery stenosis (>70% stenosis) with short segment occlusive lesions (<2 cm in length) were treated. Among 20 lesions, 3 were treated with the TurboHawk system with a protective device due to lesion calcification.	The aim of our study was to determine the usefulness of intraoperative ultrasonography (USG) during DA for evaluating the early results of this procedure. The percentage of stenosis during and after DA was determined with USG. Primary patency, which was defined as a peak systolic velocity ratio ≤3.5 with no reintervention at 6 months	Median follow-up was 5.1 months, and the procedural success rate (<30% stenosis at the end of the procedure) was 100% on angiography, but only 30% on intraoperative USG. On USG, median residual stenosis was 40% (range, 28%-42%) at the end of DA, 40% (range, 30%-55%) at 1 month, 55% (range, 35%-85%) at 6 months, and 64% (range, 60%100%) at 1 year. There was 1 dissection, but no cases of perforation, pseudoaneurysm, or thrombosis. Primary patency= in 18 lesions (90%), 11 of 14 patients (78.6%) were free of ischemic symptoms such as claudication at 6 months.	28090505
Bath J 2016; USA; (1)	All published series in the English language were identified through a systematic PubMed search. Standard descriptive statistics, reported as mean ± SD, were applied to perform the pooled analysis and calculate the overall outcome measures. Combined overall effect sizes were calculated using fixed-effect meta-analysis.	Endovascular interventions to the common femoral artery and common femoral artery can be performed safely with high technical success. Endovascular therapy may be a favored approach over endarterectomy for highly selected	The analysis included 20 studies with a total of 836 patients (897 limbs, mean age of 70.5 ± 4.3 years, critical limb ischemia 39.6%). Technical success was 95%. Angioplasty alone was undertaken in 68.8% of cases and stenting in 22.3%. Access complications occurred in 2.4% of cases. Post-operative major adverse limb events	26346964

			occurred in 2% and major adverse cardiovascular events in 1% of cases. Primary patency at 6, 12 and 24 months was 87%, 77% and 73%, respectively. Subgroup analysis revealed a significantly higher mean primary patency at 12 months for routine stenting compared to a selective stenting strategy (91.4% versus 75%; $p < 0.05$).	
Klein AJ 2014 USA (1)	Successful endovascular intervention for femoral-popliteal (FP) arterial disease provides relief of claudication and offers limb-salvage in cases of critical limb ischemia. Technologies and operator technique have evolved to the point where we may now provide effective endovascular therapy for a spectrum of lesions, patients, and clinical scenarios.	Endovascular treatment of this segment offers a significant alternative to surgical revascularization, and may confer improved safety for a wide range of patients, not solely those deemed high surgical risk. Although endovascular therapy of the FP segment has historically been hampered by high rates of restenosis, emerging technologies including drug-eluting stents, drug-coated balloons, and perhaps bio-absorbable stent platforms, provide future hope for more durable patency in complex disease.	By combining lessons learned from clinical trials, international trends in clinical practice, and insights regarding emerging technologies, we may appropriately tailor our application of endovascular therapy to provide optimal care to our patients. This document was developed to guide physicians in the clinical decision-making related to the contemporary application of endovascular intervention among patients with FP arterial disease.	24753020
Katsanos K 2014 London,UK Standards of Practice for Superficial Femoral and Popliteal Artery Angioplasty and Stenting (1)	This is a Standards of Practice document endorsed by CIRSE. The authors performed a literature review and provide recommendations and quality improvement guidelines based on the most recent and highest level of evidence available to date on the field of Superficial Femoral and Popliteal Artery Angioplasty and Stenting.	Standards for the use of basic and more advanced endovascular techniques in the femoropopliteal arteries are reported and relevant aspects of case selection, imaging, follow-up, and overall patient management are presented to guide endovascular practice in Europe.	This document was developed to guide physicians in the clinical decision-making related to the contemporary application of endovascular intervention among patients with FP arterial disease.	24722891
Antoniou GA 2008 Greece systematic review (3)	to analyze the indications, technical limitations and the outcome of remote SFA endarterectomy (RSFAE). The English literature was searched using the MEDLINE elec-	Average primary and secondary patency rates Search identified 19 retrospective or prospective case series; no randomized controlled trials comparing RSFAE with	The weighted mean cumulative primary patency rates were 60%, 57% and 35% at 1, 2 and 5 years, respectively. The weighted mean assisted primary patency rates were 75%, 77% and 50% at 1, 2 and 5 years, respectively. The	18538596

	tronic database up to February 2008. We considered studies comprising at least 10 patients treated with RSFAE and reporting on the primary and/or secondary patency rates.	another treatment modality were identified. The average technical success rate was 94% and the procedure-related complication rate was 14.7%.	weighted mean secondary patency rates were 88% and 62% at 1 and 2 years, respectively.	
Derksen WJ 2008 Netherlands (1)	Remote superficial femoral artery endarterectomy (RSFAE) is an effective minimal invasive treatment modality of TransAtlantic Inter-Society Consensus (TASC) C and D atherosclerotic lesions of the superficial femoral artery (SFA) with at least equal patency rates as supragenicular synthetic bypass grafts.	The intimal core distally of the transaction zone is secured by an expandable polytetrafluoroethylene-covered nitinol stent. By its minimal invasive character, RSFAE will lead to lower rate of post-operative complications and shorter hospital stay compared to supragenicular bypass graft surgery.	Achilles heel of RSFAE is the relatively high percentage of first year restenosis due to neointimal hyperplasia. Strict follow-up at 3, 6 and 12 months is advised including duplex ultrasound. In case of symptomatic or asymptomatic hemodynamic restenosis (>50%) percutaneous transluminal angioplasty must be performed to improve long-term patency.	18431339
Gabrielli R 2012 Rome, Italy Randomized controlled trial (2)	From October 2004 to December 2008, 95 patients with TransAtlantic Inter-Societal Consensus (TASC) D lesions were randomized 1:1 to receive RE of the superficial femoral artery (SFA) with end point stenting (51 patients) or ENDO, consisting of subintimal angioplasty with stenting (44 patients)	Primary patency was 76.5% (39 of 51) in RE and 56.8% (25 of 44) in ENDO (hazard ratio [HR], 2.6; 95% CI, 0.99-4.2; P=.05) at 24 months and was 62.7% (32 of 46) in RE and 47.7% (21 of 40) in ENDO (HR, 1.89; 95% CI, 0.94-3.78; P=.07) at 36 months.	Assisted primary patency was 70.6% (36 of 51) in RE and 52.3% (23 of 44) in ENDO (HR, 2.45; 95% CI, 1.20-5.02; P=.01). Secondary patency overlapped the primary comparison data at 12 and 24 months; at 36 months, there was a slight but significative advantage for RE (HR, 2.26; 95% CI, 1.05-4.86; P=.03).	23044257
Gisbertz SS 2010 Netherlands randomized (2)	To investigate the optimal surgical treatment, remote superficial femoral artery endarterectomy (RSFAE) or supragenicular bypass, for Transatlantic Inter-Society Consensus (TASC) C and D lesions of the superficial femoral artery. The study randomized 116 patients, 61 to RSFAE and 55 to supragenicular bypass surgery.	Medium-term results will be presented.	Primary patency after 3 years of follow-up was 47% for RSFAE and 60% for bypass (p = 0.107), assisted primary patency was 63 and 69% (p = 0.406), and secondary patency was 69 and 73% (p = 0.541), respectively. For venous (n = 25) and prosthetic grafts (n = 30) at 3-year follow-up, primary patency was 65% and 56 versus 47% for RSFAE (p = 0.143), assisted primary patency was 84% and 56 versus 63% for RSFAE (p = 0.052), and secondary patency was 89% and 59 versus 69%	21035693
Bracale UM 2016 Italy prospective (3)	(TurboHawk/Covidien-ev3 Endovascular Inc., North Plymouth, Minnesota, USA) for the treatment of femoro-popliteal obstructive disease. Patients were evaluated at 12 months.	one year, in the percutaneous treatment of femoropopliteal obstructive disease in patients with critical limb ischemia.	all patients. Clinical (Rutherford class improvement) and hemodynamic success (Ankle-brachial index improvement) was achieved in all patients.	27896226

Babaev A 2016 USA prospectively (3)	13 consecutive patients who developed Angio-Seal-associated femoral artery occlusions that occurred from 3 hours to several weeks after catheterization. These patients were successfully treated with TurboHawk /HawkOne directional atherectomy followed by balloon angioplasty with no complications.	We describe a novel approach for the endovascular treatment of femoral artery occlusion as a result of Angio-Seal closure device deployment.	During a mean follow-up period of 20.4 ± 17.3 months, 12 patients remained claudication free with no evidence of obstructive arterial disease of the treated segment on imaging studies. 1 patient developed restenosis that was treated with repeat atherectomy and balloon angioplasty following which he was asymptomatic at followup.	27852881
Stavroulakis K 2015 Germany prospective study (3)	between October 2009 and February 2014 21 patients (mean age 63±16 years; 16 men) with isolated popliteal artery lesions were enrolled and un-derwent treatment with combined DA and DEB angioplasty under filter pro-tection. The majority (18, 86%) pre-sented with life-style-limiting intermit-tent claudication and 3 with critical limb ischemia. 15 (71%) target sites were de novo lesions; 4 were occlusions.	the midterm results of com-bined di-rectional atherec-tomy (DA) and drug-eluting balloon (DEB) angioplasty for atherosclerotic lesions of the popliteal artery. The main outcome was pri-mary patency; secondary outcomes were technical success, secondary patency, and early and midterm morbid-ity and mortality.	The TurboHawk atherectomy device was used in 15 (71%) patients and the SilverHawk peripheral plaque excision system in the remaining 6 patients. The technical success rate was 90% (n=19). One flow-limiting dissection was treated with bailout stenting. Complications in-cluded a perforation of the popliteal artery and 2 puncture site hematomas; there was no distal embolic event. The mean follow-up was 18±12 months. 2 restenoses were retreated success-fully. Kaplan-Meier estimates of primary pa-tency at 12 and 18 months were 95% and 90%, respectively; the secondary patency was 100%. 1 patient (5%) died in follow-up. None of the patients had an amputation.	26420802
Roberts D 2014 USA the DEFINITIVE Ca(++) study; prospectively (3)	A total of 133 subjects with 168 moderate to severely calcified lesions were enrolled. Lesions were treated with directional atherectomy devices, coupled with distal embolic protection.	evaluate the safety and effectiveness of directional atherectomy and distal embolic protection, used together to treat moderate to severely calcified femoropopliteal lesions.	The 30-day freedom from MAE rate was 93.1%. Per angiographic assessment, the primary effectiveness endpoint (≤50% residual diameter stenosis) was achieved in 92.0% (lower confidence bound of 87.6%) of lesions. A mean residual diameter stenosis of 33.3% was achieved with the directional atherectomy device. This was further decreased to 24.1% with the use of adjunctive therapy. The proportion of asymptomatic subjects [Rutherford Clinical Category (RCC) = 0] increased from 0% at baseline to 52.3% at the 30-day follow-up visit. In total,	24402764

			88.5% of subjects experienced an improvement of one or more Rutherford categories.	
Rosenthal D 2006 USA prospectively (3)	A total of 210 patients were included in this study. The indications for the procedure were claudication in 158 (75%) patients and limb salvage in 52 (25%). After RSFAE the outflow tract atheromatous plaque was "tacked" with the aSpire stent, which is an expanded polytetrafluoroethylene (ePTFE) covered nitinol stent with high radial strength, yet it is flexible enough to withstand the compressive forces at the knee joint. Prior to stent deployment, if the stent position is not in optimal position, it can be "wrapped down", repositioned and reexpanded.	The aim of this study was to examine the results of remote superficial femoral artery endarterectomy (RSFAE) in conjunction with distal aSpire stenting in a multinational study. RSFAE is a minimally invasive procedure performed through a limited groin incision.	The mean length of endarterectomized superficial femoral arteries (SFAs) was 28.2+/-6.2 cm (range 15-43 cm). The primary cumulative patency rate by means of life-table analysis was 60.6+/-4.8% (SE) at 33 months, (mean 17.1 months; range 1-33 months). During follow-up percutaneous transluminal balloon and/or stent angioplasty was necessary in 50 patients for a primary assisted patency of 70.2+/-4.8% at 33 months. The locations of the restenosis after RSFAE were evenlydistributed along the endarterectomized artery. There were 2 deaths (myocardial infarctions), 12 (5.7%) wound complications (7 hematomas, 5 skin edge sloughs) and the mean hospital length of stay was only 1.3+/-0.5 days.	16953157
Knight JS 2005 UK. Prospective multi-center trial (3)	62 limbs in 61 patients (41 men; median age 69 years, range 40-88) with severe disabling claudication (n=56) or critical limb ischaemia (n=6) were treated in five European centres with aSpire stenting after RSFAE for long segment occlusions (mean length 25 cm). Follow-up was by duplex scanning at 1-, 6-, 12- and 18-months.	To evaluate the feasibility and efficacy of an stent and adjustable deployment system (aSpire Covered Stent, Vascular Architects Inc., San Jose, CA, USA) in combination with RSFAE for the treatment of long segment femoropopliteal	The median follow-up was 17 (range 2-34) months. A mean of 1.3 stents (range 1-3) were deployed with a median stent diameter of 7 mm (range 6-9). There were 1 early and 24 late failures. At 18-months the cumulative primary, primary-assisted and secondary patency rates were 60, 70 and 72%, respectively. There were no device related adverse events, such as kinking or fracturing and no stent migrations.	15694803
Rosenthal D 2004 USA (3)	RSFAE is a minimally invasive procedure performed through a limited groin incision. 40 patients were included in the study.	Examine the results of remote superficial femoral artery endarterectomy	The primary cumulative patency rate by means of life table analysis was 68.6% +/- 13.5% (SE) at 18 months (mean, 13.2 months; range, 1-31 months).	15218464
Werner-Gibbings K 2014	To assess the efficacy and safety of excisional atherectomy with the TurboHawk atherectomy device in the treatment of lower limb peripheral vascular disease. Patients undergoing	Endovascular interventions are increasingly utilized in managing occlusive peripheral vascular disease. Angioplasty and stenting remain the	Atherectomy was possible in 98% of cases. The 6- and 12-month primary patency was 72.6 and 58.9%, respectively. The primary-assisted patency was 93.2% at 6 months and 74.6% at 12	25366124

Australia retrospective analysis (4)	atherectomy for symptomatic lower limb peripheral vascular disease by a single clinician between 2011 and June 2013: 47 vessels on 28 legs in 24 patients were treated.	mainstay of endovascular management; however, newer treatment modalities such as excisional atherectomy.	months. There were 4 instances of embolization and 4 cases of dissection.	
Rosenthal D 2001 USA retrospective multicenter study (4)	Remote superficial femoral artery endarterectomy (RSFAE) is a minimally invasive procedure, performed through a single limited groin incision that may offer patency rates comparable with those of above-knee femoropopliteal (AKFP) bypass graft. 60 patients were included in this study. Indications for the procedure were claudication in 52 patients and limb salvage in eight patients.RSFAE was performed with the MollRing Cutter device through a femoral arteriotomy. The distal "flap" of atheroma was anchored by balloon/stentangioplasty through the femoral arteriotomy.	the medium-term results of all patients underwent a follow-up examination with serial color flow ultrasound scanning.	10 patients with heavily calcified SFAs failed as "intentions to treat"; these patients underwent AKFP bypass grafting. The mean length of the endarterectomized SFAs was 22.3 cm (range, 837 cm). The primary cumulative patency rate by means of life-table analysis was 61.4% +/- 9% (SE), (mean, 12.9 months; range, 3-36 months). During follow-up, percutaneous transluminal angioplasty was necessary in 14 patients, for a primary-assisted patency rate of 82.6% +/- 8%. The locations of the restenoses after RSFAE were evenly distributed along the endarterectomized SFAs. There were no deaths and 1 wound complication (hematoma), and the mean hospital length of stay was 1.4 days +/- 0.8 days.	11533593
Sheng N 2014 USA retrospective review of a prospectively maintained database (4)	from January 2009 to September 2011 21 patients underwent RSFAE at our institution. The study group was com-prised of 5 patients undergoing RSFAE and adjunct distal revasculari-zation for critical limb ischemia dur-ing the study period. 4 patients (80%) presented with tissue loss, and 1 (20%) presented with ischemic rest pain. 3 (60%) required simultaneous outflow sequential vein bypass and 2 (40%) required distal endovascular re-vascularization.	Outcome variables, includ-ing patency, time to heal-ing, limb salvage, ambula-tory status, and survival, were analyzed.	Technical success was 100%, and there were no early reconstruction failures. There was 1 pop-liteal wound complication, and no groin wound complications during the study follow-up. At 6 months postoperatively, 4 of 5 reconstructions were patent. 2 of 5 patients (40%) required per-cutaneous reintervention for restenosis at 10 and 11 months, respectively. Primary assisted pa-tency was 80% with a mean follow-up of 12.6 months (range 8-22 months). The limb salvage rate was 80% and there have been no deaths.	24084265

Tabarin AS, Chupin AV 2010 Russia review (5)	The review contains the literature data con-cerning various types of in-frainguinal inter-ventions in patients presenting with fem-oropopliteal occlusion: balloon angioplasty and stenting, femoropopliteal bypass graft-ing, remote endarterectomy, etc.	Special mention should be made of treating the pa-tients with critical is-chae-mia. The problem of choos-ing between surgical and endovascular treatment for occlusive stenotic dam-age to the superficial femoral artery is featured insuffi-ciently in the present-day literature.	Further studies should be conducted.	21780636
Garcia LA 2015 USA, Germany. US/European multicenter study (3)	DEFINITIVE LE, a US/European multicenter study, assessed the effectiveness of direc-tional atherectomy using SilverHawk/Turbo-Hawk systems for treatment of peripheral ar-tery disease in the superficial femoral, poplit-eal, and infrapopliteal arteries. Of the 800 pa-tients enrolled in the study, only the 598 clau-dicant patients (mean age 69.5±10.4 years; 336 men) who were classified at baseline as Rutherford category 1-3 were eligible for this subset analysis. Of these, 46.8% (280/598) had diabetes. Follow-up to 12 months.	report a subset analysis that evaluated the hypothesis that directional atherectomy for peripheral artery dis-ease in diabetic claudicants has nonin-ferior primary patency at 12 months compared with nondiabetic claudi-cants.	12-month primary patency (77.0%) was no dif-ferent than for nondiabetics (77.9%; superiority p=0.98; noninferiority p<0.001) across all ana-tomic territories treated. Freedom from clinically driven target lesion revascularization was no dif-ferent between diabetics (83.8%) and nondiabet-ics (87.5%) overall (p=0.19) or by lesion loca-tions. Secondary clinical outcomes (Rutherford category, ankle-brachial index, and walking im-pairment) improved at 12 months for both dia-betics and nondiabetics.	26250748
McKinsey JF 2014 USA, Ger-many prospec-tively (3)	DEFINITIVE LE (Determination of EFfec-tiveness of the SilverHawk(®) PerIpheral Plaque ExcisioN System (SIlverHawk De-vice) for the Treatment of Infrainguinal VEssels / Lower Extremities) prospectively enrolled 800 subjects at 47 multinational centers with an infrainguinal lesion length up to 20 cm.	assess the safety and effectiveness of di-rectional atherectomy (DA) for endovas-cular treatment of peripheral arterial dis-ease (PAD) in infrainguinal arteries in patients with claudication or critical limb ischemia. Primary endpoints were defined as primary patency at 12 months for claudicants and freedom from major unplanned amputa- tion for critical limb ischemia (CLI) subjects.	The 12-month primary patency was 78% (95% confidence interval: 74.0% to 80.6%) in clau-dicants, with a 77% rate in the diabetic sub-group versus 78% in the nondiabetic subgroup (noninferior, p < 0.001). The rate of freedom from major unplanned amputation of the target limb at 12 months in CLI subjects was 95% (95% confidence interval: 90.7% to 97.4%). Periprocedural adverse events included embo-lization (3.8%), perforation (5.3%), and abrupt closure (2.0%). The bail-out stent rate was 3.2%.	25147039
Werner-Gibbings K 2017; Aus-	between November 2011 and June 2013. Forty-seven vessels on 28 legs in 24 pa-tients were treated during the period.	Excisional atherectomy provides a further option for the minimally inva-	Atherectomy was possible in 98% of cases. The 6- and 12-month primary patency was 72.6 and	25366124

tralia; A retrospective analysis (4)		sive management of peripheral vascular disease. It has similar patency rates to established endovascular therapies and should be considered among the treatment options in patients with favourable pathology.	58.9%, respectively. The primary-assisted patency was 93.2% at 6 months and 74.6% at 12 months. There were significantly greater patency rates in the TransAtlantic Inter-Society Consensus A + B lesions and a non-significant trend towards improved patency rates in claudicants versus critical limb ischaemia. There were four instances of embolization and four cases of dissection.	
Quevedo HC 2014 USA review (5)	A database search for "directional," "orbital," "rotational," and "laser atherectomy" in peripheral arterial disease (PAD) was performed.	To assess the impact of atherectomy on *primary patency* rates at 12 months compared to balloon angioplasty and/or stent placement alone in patients with infrainguinal arterial disease. primary patency and major adverse events	Only 2 randomized studies were found. Most of the data were obtained from single-arm studies and registries. The primary patency with *directional atherectomy* approaches 60% at 12 months as a standalone technique, whereas *orbital atherectomy* in conjunction with balloon angioplasty and stenting achieved primary patency rates of 90%. *Laser atherectomy* is universally employed with balloon angioplasty and stenting for in-stent restenosis lesions with a primary patency rate of 64%.	24402808
Lensvelt MM 2012 Netherlands (5)				22433735
Shafique S 2007; USA; (5)	There are multiple endovascular options to achieve percutaneous revascularization of chronic superficial femoral artery (SFA) stenoses and occlusions.	Remote SFA endarterectomy with the Aspire stent (Vascular Architects, San Jose, CA) is a hybrid surgical and endovascular technique that is useful for debulking plaque from the SFA with adjunctive stenting of the distal SFA	Currently, directional atherectomy is performed using the Silverhawk Plaque Excision System, while laser atherectomy is frequently performed.	17386361
Moll FL 1999 Netherlands (5)	Semiclosed endarterectomy of the SFA belongs in the armamentarium of the vascular surgeon.	New technology offers the possibility of performing this less invasive operation so that only a single incision is needed to obtain access to the artery and perform remote disobliteration.	Strong indications show that the anticipated restenosis of long, segmental, closed endarterectomies can be reduced remarkably by expanded PTFE endolining	10410690

Table S2. The main conclusions based on the Best evidence from studies included in the qualitative -synthesis

First author, date, country of research, study type (and LoE)	Any identified study weaknesses or conclusions
Klein AJ 2014. [7]<br>USA<br>(1)	Successful endovascular intervention for femoral-popliteal (FP) arterial disease provides relief of claudication and offers limb-salvage in cases of critical limb ischemia. Endovascular treatment of this segment offers a significant alternative to surgical revascularization, and may confer improved safety for a wide range of patients, not solely those deemed high surgical risk.
Katsanos K 2014. [8]<br>London,UK<br>Standards of Practice for Superficial Femoral and Popliteal Artery Angioplasty and Stenting (1)	An Endovascular-First Approach is recommended in the majority of femoropopliteal stenoses or occlusions, although vein bypass surgery still has a role in case of long or heavily calcified chronic total occlusions (CTO) and in patients with a favourable life-expectancy. Intraluminal or subintimal recanalization techniques may be used based on operator preference and experience. RCTs have also shown marginally increased primary patency rates following primary nitinol stent placement. Evidence about the use of drug-eluting stents (DES) has been conflicting, although they do seem to decrease the rate of repeat procedures compared with plain balloon angioplasty. Covered stents inhibit neointimal ingrowth and seem to perform similar to nitinol stents. Paclitaxel-coated balloons have been show to outperform balloon angioplasty in several, randomized, controlled trials, but long-term evidence is still missing. Unmet needs for treatment include in-stent restenosis and the implantable foreign material if stenting is performed. Bioabsorbable vascular scaffolds may address the latter in the future. All patients should receive at least single antiplatelet therapy following a successful endovascular procedure. Overall, this is a rapidly evolving field with several ongoing studies, and operators need to remain up to date with the literature.
Antoniou GA 2008. [9]<br>Greece<br>systematic review (3)	Remote endarterectomy is a minimally invasive procedure which combines open and endovascular surgery for the treatment of long segment superficial femoral artery (SFA) occlusive disease. RSFAE has acceptable short-, medium- and long-term results but patients should undergo intensive surveillance postoperatively.<br>RCTs are needed to assess the durability of this procedure as compared to conventional open bypass surgery.
Derksen WJ 2008. [10]<br>Netherlands<br>(1)	Remote superficial femoral artery endarterectomy (RSFAE) is an effective minimal invasive treatment modality of Trans-Atlantic Inter-Society Consensus (TASC) C and D atherosclerotic lesions of the superficial femoral artery (SFA) with at least equal patency rates as supragenicular synthetic bypass grafts. This procedure is performed through a single femoral arteriotomy and the intima core in the SFA is dissected using the Vollmar ring and the Mollring cutter devices, respectively. The intimal core distally of the transaction zone is secured by an expandable polytetrafluoroethylene-covered nitinol stent. By its

	minimal invasive character, RSFAE will lead to lower rate of postoperative complications and shorter hospital stay compared to supragenicular bypass graft surgery. Reobstruction of the SFA tends to have, in contrast to bypass grafts, less severe symptoms due to preservation of collaterals and thereby lower amputation rate. Achilles heel of RSFAE is the relatively high percentage of first year restenosis due to neointimal hyperplasia. Strict follow-up at 3, 6 and 12 months is advised including duplex ultrasound. In case of symptomatic or asymptomatic hemodynamic restenosis (>50%) percutaneous transluminal angioplasty must be performed to improve long-term patency. The majority of reobstructions can be treated by endovascular means.
Gabrielli R 2012. [11]<br>Rome, Italy<br>Randomized controlled trial (2)	Remote endarterectomy (RE) of the superficial femoral artery (SFA is a safe, effective, and durable procedure for TASC-II D lesions. Our data demonstrate a significantly higher primary, assisted primary, and secondary patency of RE vs endovascular (ENDO) interventions procedures. RE should be considered better than an endovascular procedure in SFA long-segment occlusion treatment.
Gisbertz SS 2010. [12]<br>Netherlands randomized (2)	Venous bypass grafting is superior to both RESFA and polytetrafluoroethylene grafting, but only 45% of patients had a sufficient saphenous vein available. If the saphenous vein is not applicable, RSFAE should be considered because it is less invasive and prosthetic graft material can be avoided.
Bracale UM 2016. [13]<br>Italy<br>prospective (3)	Femoro-popliteal PTA for the treatment of critical limb ischemia is frequently associated with unsatisfactory procedural success rates while directional atherectomy (DA) has improved success rate since claudicant patients undergoing percutaneous treatment of femoro-popliteal obstructive disease. The use of DA for the treatment of femoro-popliteal obstructive disease is a safe and effective therapeutic strategy for patients with critical limb ischemia.
Babaev A 2016. [14]<br>USA<br>prospectively (3)	Angio-Seal is the most commonly used vascular closure device following percutaneous coronary and peripheral catheterizations worldwide. A rare complication of Angio-Seal deployment is an occlusion of the femoral artery leading to limb ischemia requiring revascularization. Given its unique ability to cut both atherosclerotic plaque and the Angio-Seal anchor with a collagen plug at operator-directed planes, TurboHawk/HawkOne atherectomy device can be a fast and effective approach to treat Angio-Seal-associated femoral artery occlusions. The use of directional atherectomy followed by balloon angioplasty is a quick, safe, and effective endovascular approach to treating Angio-Seal-associated femoral artery occlusions. It is associated with an excellent success rate, no complications, and good midterm outcomes.
Stavroulakis K 2015. [15]<br>Germany<br>prospective study (3)	In this prospective single-arm study, the combined therapy of DA and DEB angioplasty for popliteal artery lesions showed promising midterm performance. The combination of DA and DEB may, in highly selected patients, overcome the challenges presented by the mobility of the knee joint.

Sheng N 2014. [16]<br>USA<br>retrospective review (4)	In an effort to avoid the use of prosthetic material, we evaluated the use of RSFAE with distal autogenous revascularization in patients with critical limb ischemia and limited conduit. Further study is needed to determine whether the long-term results are superior to distal composite bypass or polytetrafluoroethylene bypass alone.
Roberts D 2014. [17]<br>USA<br>prospectively (3)	The results of the DEFINITIVE Ca++ study demonstrate that the SilverHawk and TurboHawk atherectomy devices are safe and effective in the endovascular treatment of moderate to severely calcified lesions in the superficial femoral and/or popliteal arteries when used with the SpiderFX distal embolic protection device.
Rosenthal D 2004. [18]<br>USA<br>(3)	The advent of minimally invasive procedures such as percutaneous transluminal angioplasty with or without stent, laser-assisted balloon angioplasty, and atherectomy, whose results have proven disappointing in the treatment of long-segment (> more than 15 cm) superficial femoral artery (SFA) occlusive disease, stimulated a reassessment of SFA endarterectomy. With the evolution of remote superficial femoral artery endarterectomy (RSFAE) a minimally invasive technique became available which could be performed through a single incision, allowed, debulking of the arterial plaque, and placement of an endovascular stent.
Knight JS 2005. [19]<br>UK.<br>Prospective multi-centre (3)	The aSpire stent and the delivery system are both safe and feasible in combination with RSFAE. The midterm follow-up appears favourable in view of the long segment occlusions treated. Further follow-up is required to compare the mid- and long-term outcomes with current stents and conventional femoropopliteal bypass.
Rosenthal D 2004. [20]<br>USA<br>prospectively (3)	RSFAE with distal aSpire stenting is a safe and moderately durable procedure. If long-term patency rates are similar to those of above-knee femoropopliteal bypass graft, this procedure may prove to be a minimally invasive adjunct for the treatment of superficial femoral artery occlusive disease.
Werner-Gibbings K 2014. [21]<br>Australia<br>retrospective analysis (4)	Excisional atherectomy provides a further option for the minimally invasive management of peripheral vascular disease. It has similar patency rates to established endovascular therapies and should be considered among the treatment options in patients with favourable pathology.
Rosenthal D 2001. [22]<br>USA<br>retrospective multicenter (4)	RSFAE is a safe and moderately durable procedure. If long-term patency rates are similar to those of AKFP bypass graft, RSFAE may prove to be a minimally invasive adjunct for the treatment of SFA occlusive disease that will lower operative morbidity, reduce hospital LOS, and shorten recuperation.
Tabarin AS, Chupin AV 2010. [23]<br>Russia<br>review (5)	The review contains the literature data concerning various types of in-frainguinal interventions in patients presenting with femoropopliteal occlusion: balloon angioplasty and stenting, femoropopliteal bypass grafting, remote endarterectomy

Garcia LA 2015. [24] USA, Germany. US/European multicenter study (3)	Noninferior 12-month patency rates demonstrate that directional atherectomy is an effective treatment in diabetic as well as nondiabetic claudicants. Directional atherectomy remains an attractive treatment option, improving luminal diameters without stents, which preserves future treatment options for both diabetic and nondiabetic patients with progressive, diffuse vascular disease.
McKinsey JF 2014. [25] USA, Germany prospectively (3)	The DEFINITIVE LE study demonstrated that DA is a safe and effective treatment modality at 12 months for a diverse patient population with either claudication or CLI. Furthermore, DA was shown to be noninferior for treating PAD in patients with diabetes compared with those without diabetes.
Werner-Gibbings 2017. [26]	
Quevedo HC 2014. [27] USA review (5)	By decreasing the volume of the atherosclerotic plaque, debulking procedures may confer superior primary patency after revascularization. Despite the successful procedural outcomes reported in clinical registries, the available data do not support the use of atherectomy alone in PAD. Larger randomized controlled studies are warranted to define its role in contemporary endovascular practice.
Lensvelt MM 2012. [28] Netherlands (5)	Although the autologous venous conduit is still considered the gold standard for treatment of long occlusive SFA lesions, endoluminal therapy is gaining territory. Percutaneous transluminal angioplasty is first choice in short SFA lesions, but patency rates decrease with longer lesions. When percutaneous transluminal angioplasty is combined with nitinol stent placement patency rates significantly improve.
Shafique S 2007. [29] USAa review of literature (5)	There are multiple endovascular options to achieve percutaneous revascularization of chronic SFA stenoses and occlusions. Most rely on forceful displacement of plaque via balloon angioplasty, either as a stand-alone therapy or supplemented by cold thermal injury (cryoplasty), microtome assistance (cutting balloon angioplasty), nitinol stent deployment, or expanded polytetrafluoroethylene-lined nitinol stent deployment. The essential problem associated with these techniques is the predictable compromise of the initial result by neointimal hyperplasia leading to poor long-term results. An alternative to forceful displacement techniques is use of directional atherectomy or excimer laser to debulk the atheromatous lesion, with the addition of low-pressure angioplasty or stent deployment as needed. Currently, Directional Atherectomy is performed using the Silverhawk Plaque Excision System (FoxHollow, Redwood City, CA). Remote SFA endarterectomy with the Aspire stent (Vascular Architects, San Jose, CA) is a hybrid surgical and endovascular technique that is useful for debulking plaque from the SFA with adjunctive stenting of the distal SFA.
Moll FL 1999. [30] Netherlands	Semiclosed endarterectomy of the SFA belongs in the armamentarium of the vascular surgeon. New technology offers the possibility of performing this less invasive operation so that only a single incision is needed to obtain access to the artery

(5)		and perform remote disobliteration. Strong indications show that the anticipated restenosis of long, segmental, closed endarterectomies can be reduced remarkably by expanded PTFE endolining.

Table S3. The results of the data extraction from studies included in the meta-analysis

Author, date, country of re search, (and LoE)	Patient group	Key results
Antoniou GA 2008 (3) [9]	Outcome of RSFAE. The English literature was searched using the MEDLINE database up to February 2008. We considered studies comprising at least 10 patients treated with RSFAE and reporting on the primary and/or secondary patency rates. Search identified 19 retrospective or prospective case series; no randomized controlled trials comparing RSFAE with another treatment modality were identified.	The average technical success rate was 94% and the procedure related complication rate was 14.7%. The weighted mean cumulative primary patency rates were 60%, 57% and 35% at 1, 2 and 5 years, respectively. The weighted mean assisted primary patency rates were 75%, 77% and 50% at 1, 2 and 5 years, respectively. The weighted mean secondary patency rates were 88% and 62% at 1 and 2 years, respectively.
Gabrielli R 2012 (2) [11]	From 2004 to 2008, 95 patients with TransAtlantic InterSocietal Consensus (TASC) D lesions were randomized 1:1 to receive remote superficial femoral artery endarterectomy (RSFAE) with end point stenting (51 patients) or ENDO, consisting of subintimal angioplasty with stenting (44 patients). –5 RSFAE patients and four ENDO patients were lost to follow up (censored). –Survival curves for primary patency were plotted using the Kaplan Meier method. –The mean follow up was 52.5 months (range, 3575 months).	Primary patency was 76.5% (39 of 51) in RSFAE and 56.8% (25 of 44) in ENDO (hazard ratio [HR], 2.6; 95% CI, 0.99 4.2; P=.05) at 24 months and was 62.7% (32 of 46) in RSFAE and 47.7% (21 of 40) in ENDO (HR, 1.89; 95% CI, 0.943.78; P=.07) at 36 months. Assisted primary patency was 70.6% (36 of 51) in RSFAE and 52.3% (23 of 44) in ENDO (HR, 2.45; 95% CI, 1.205.02; P=.01). Secondary patency overlapped the primary comparison data at 12 and 24 months; at 36 months, there was a slight but significative advantage for RSFAE (HR, 2.26; 95% CI, 1.054.86; P=.03).
Gisbertz SS 2010 (2) [12]	To investigate the optimal surgical treatment, RSFAE or supragenicular bypass, for Transatlantic InterSociety Consensus (TASC) C and D le-	Primary patency after 3 years of follow up was 47% for RSFAE and 60% for bypass (p = 0.107), Assisted primary patency was 63 and 69%

	sions of the superficial femoral artery. The study randomized 116 patients, 61 to RSFAE and 55 to supragenicular bypass surgery. Indications for surgery were claudication in 77, rest pain in 21, or tissue loss in 18.	(p = 0.406), Secondary patency was 69 and 73% (p = 0.541), respectively. –For venous (n = 25) and prosthetic grafts (n = 30) at 3year follow up, Primary patency was 65% and 56 versus 47% for RSFAE (p = 0.143), Assisted Primary Patency was 84% and 56 versus 63% for RSFAE (p = 0.052), and Secondary patency was 89% and 59 versus 69% for RSFAE (p = 0.046), respectively. Limb salvage was 97% after RSFAE and 95% after bypass surgery (p = 0.564).
Sheng N 2014 (4) [16]	from 2009 to 2011 5 patients undergoing RSFAE and adjunct distal revascularization. 3 (60%) required simultaneous outflow sequential vein bypass and 2 (40%) required distal endovascular revascularization.	Technical success was 100%. At 6 months postoperatively, 4 of 5 reconstructions were patent. 2 of 5 patients (40%) required percutaneous reintervention for restenosis. Primary assisted patency was 80% with a mean follow up of 12.6 months (range 822 months). The limb salvage rate was 80% and there have been no deaths.
Rosenthal D 2006 (3) [18]	A total of 210 patients were included in this study. The indications for the procedure were claudication in 158 (75%) patients and limb salvage in 52 (25%). After RSFAE the outflow tract atheromatous plaque was "tacked" with the stent. The mean length of endarterectomized superficial femoral arteries (SFAs) was 28.2+/6.2 cm (range 1543 cm).	The primary cumulative patency rate was 60.6+/4.8% (SE) at 33 months, (mean 17.1 months; range 133 months). During follow up percutaneous transluminal balloon and/or stent ang ioplasty was in 50 patients for a primary assisted patency of 70.2+/4.8% at 33 months. There were 2 deaths (myocardial infarctions), 12 (5.7%) wound complications (7 hematomas, 5 skin edge sloughs) and the mean hospital length of stay was only 1.3+/0.5 days.
Knight JS 2005 (3) [19]	To evaluate the feasibility and efficacy of an stent and adjustable deployment system (aSpire Covered Stent, Vascular Architects Inc., San Jose, CA, USA) in combination with RSFAE for the treatment of long segment femoro popliteal	The median follow up was 17 (range 234) months. A mean of 1.3 stents (range 13) were deployed with a median stent diameter of 7 mm (range 69). There were 1 early and 24 late failures. At 18months the cumulative primary, primary assisted and secondary patency rates were 60, 70 and 72%, respectively. There were no device related adverse events, such as kinking or fracturing and no stent migrations.

Garcia LA 2015 (3) [24]	DEFINITIVE LE, a US/European multicenter study, assessed the effectiveness of DA using SilverHawk/TurboHawk systems for treatment of peripheral artery disease in the superficial femoral, popliteal, and infrapopliteal arteries. 598 claudicant patients (mean age 69.5±10.4 years; 336 men) who were classified at baseline as Rutherford category 13. Of these, 46.8% (280/598) had diabetes. Follow up to 12 months.	12month primary patency (77.0%) was no different than for non,/diabetics. Freedom from clinically driven target lesion revascularization was no different between diabetics (83.8%) and nondiabetics (87.5%) or by lesion locations. Secondary clinical outcomes improved at 12 months for both diabetics and nondiabetics.
McKinsey JF 2014 (3) [25]	DEFINITIVE LE (Determination of EFfectiveness of the SilverHawk(®) PerIpheral Plaque ExcisioN System (SIlverHawk Device) for the Treatment of Infrainguinal VEssels / Lower Extremities) prospectively enrolled 800 subjects at 47 multinational centers with an infrainguinal lesion length up to 20 cm.	The 12month primary patency was 78% (95% CI: 74.0% to 80.6%) in claudicants, with a 77% rate in the diabetic subgroup versus 78% in the nondiabetic subgroup (noninferior, p < 0.001). The rate of freedom from major unplanned amputation of the target limb at 12 months in CLI subjects was 95% (95% CI: 90.7% to 97.4%). Periprocedural adverse events included embolization (3.8%), perforation (5.3%), and abrupt closure (2.0%). The bailout stent rate was 3.2%.

Table S4. The main data based on the Best evidence from studies included in the indirect meta-analysis

(total **9 studies**; Level of Evidence, Oxford 2011 (LoE: 1/2/3/4/5), = **0/2/5/2/0**; total **2762 patients**)

**Author, date, (and LoE)**	**Total patients**	**Events (expected):** (primary patency/obstruction)	**Key results:**	**“Primary patency”, mean (and LoE)**
1.Group S/TH, Events; total 3 studies (LoE: 1/2/3/4/5 = 0/0/2/1/0, respectively); total **1422 patients**:				
[24]Garcia LA 2015 (3)	598	460/138	12-month primary patency (77.0%)	77,5% (3)
[25]McKinsey JF 2014 (3)	800	624/176	The 12-month primary patency was 78%	
[26]Werner-Gibbings K 2014 (4)	24	14/10	The 12-month primary patency was 58.9%	58.9% (4)

2.Group RSFAE, Controls; total 6 studies (LoE: 1/2/3/4/5 = 0/2/3/1/0, respectively); total **1340 patients**:

[9]Antoniou GA 2008 (3)	952	571/381	1 year primary patency rates were 60%	
[11]Gabrielli R 2012 (2)	51	40/11	The primary 12-month patency rate was 78.4%	71.2% (2)
[12]Gisbertz SS 2010 (2)	61	39/22	Primary patency after 1 year was 64% (Kaplan-Meier survival estimate)	
[16]Sheng N 2014 (4)	5	4/1	12.6 months Primary assisted patency was 80%	80% (4)
[18]Rosenthal D 2006 (3)	210	179/32	The 12 months primary cumulative patency rate was 85%	
[19]Knight JS 2005 (3)	61	40/21	At 12-months the cumulative primary 65.4%	70.1% (3)
